# Influence of zirconium carbide particles on the mechanical characteristics of heat treated Al7475 alloy

**DOI:** 10.1038/s41598-025-99221-3

**Published:** 2025-04-29

**Authors:** Vijaya Kumar R., Asha P. B., Md. Abdul Raheem Junaidi, Jagadeesha T., Raju K., Dayanand M. Goudar, Deesy G. Pinto, Srinidhi Acharya S. R., Subraya Krishna Bhat, Shashidhar L. C., Hemanth Raju T., Udayashankar S.

**Affiliations:** 1 Aeronautical Engineering Department, MVJ College of Engineering (Affiliated to VTU), Bangalore, 560067 Karnataka India; 2 Mechanical Engineering Department, Global Academy of Technology (Affiliated to VTU), Bengaluru, 560098 Karnataka India; 3 Mechanical Engineering Department, Muffakham Jah College of Engineering & Technology, Hyderabad, 500034 Telangana India; 4 Mechanical Engineering Department, National Institute of Technology, Calicut, 673601 Kerala India; 5 Department of Mechanical Engineering, St. Joseph Engineering College, Mangaluru, 575028 India; 6 Department of Mechanical Engineering, Tontadarya College of Engineering, Gadag, 582101 India; 7 GeoBioTec, Department of Civil Engineering & Architecture, University of Beira Interior, Calcada Fonte do Lameiro 6, Covilha, 6200-358 Portugal; 8 Mechanical Engineering Department, B M S Institute of Technology, Bengaluru, 560064 Karnataka India; 9 Department of Mechanical and Industrial Engineering, Manipal Institute of Technology, Manipal Academy of Higher Education, Manipal, 576104 Karnataka India; 10 Mechanical Engineering Department, Dayananda Sagar University, Ramanagara, Bengaluru, 562112 Karnataka India; 11 Mechanical Engineering Department, New Horizon College of Engineering, Bangalore, 560103 Karnataka India; 12 Department of Mechanical Engineering, VTU, Belgaum, 590018 Karnataka India; 13 Department of Civil Engineering & Geology, University of Madeira, Campus da Penteada, Funchal, 9020-105 Portugal

**Keywords:** Al7475 alloy, Zirconium carbide (ZrC), Heat treatment, Compression strength, Hardness, Engineering, Materials science

## Abstract

Aluminium alloys exhibits excellent properties and therefore they are used broadly in automobile, aviation, and defence sectors. The studies on the effect of zirconium carbide (ZrC) particulates on the mechanical properties of heat-treated stir-cast Al7475 alloy are still in the initial stage. Hence, the present study is aimed at the study of microstructure and mechanical characteristics of as-stir-cast and heat-treated stir-cast Al7475-ZrC composites. The zirconium carbide particulates concentration ranges from 2 to 8 wt% in Al7475 alloy. The Al7475 alloy and Al7475-ZrC composites were T6 heat-treated. Both the Al7475 alloy and the Al7475-ZrC composites have undergone significant microstructure refinement owing to heat treatment. The SEM micrographs of heat-treated stir-cast Al7475-ZrC composites have revealed that the matrices of Al7475 composites consisted of a fine dispersion of uniformly distributed ZrC particles that eventually resulted in a considerable improvement in the properties of composites. The mechanical properties of heat-treated stir-cast Al7475-ZrC composites were superior to that of as-stir-cast Al7475-ZrC composites. The optimum values of UTS of as-stir-cast and heat-treated stir-cast Al7475-ZrC composites are 104.42 N/mm^2^ and 121.95 N/mm^2^, correspondingly. The optimum values of compression strength of as-stir-cast and heat-treated stir-cast Al7475-ZrC composites are 665.43 N/mm^2^ and 789.68 N/mm^2^, correspondingly. The optimum values of the hardness of as-stir-cast and heat-treated stir-cast Al7475-ZrC composites are 103.74 BHN and 126.86 BHN, respectively. The optimum values of impact strength of as-stir-cast and heat-treated stir-cast Al7475-ZrC composites are 16 J/mm^2^ and 19 J/mm^2^, respectively. Among heat-treated stir-cast composites, Al7475-6%ZrC composite has the highest enhancement in mechanical characteristics, and the UTS, compression strength, and hardness are 15%, 16%, and 18% higher than that of as-stir-cast Al7475-6%ZrC composite and 19%, 14% and 10% higher than that of heat-treated stir-cast Al7475 alloy respectively.

## Introduction

The ongoing development of new composite materials is focused on improving sustainability, mechanical properties, and integrating smart technologies to meet the evolving needs of various industries. Composite materials are manmade substances composed of two or more basic materials that exhibit markedly distinct physical or chemical characteristics. When mixed together, these ingredients create a composite with different properties from the individual ingredients. This makes composites very versatile and enables them to address a wide variety of design and functional needs in diverse applications^1^. Metal Matrix Composites (MMCs) have recently grown significant in various industrial sectors due to their good specific toughness, rigidity, and thermal resistance. When the characteristics of the base metal are desired to be improved, reinforcements are typically used. Aluminum alloy based MMCs are employed in many applications such as brake discs, cylinders, and pistons^2^.

When it comes to resistance to fatigue crack propagation, Al7475 is a material that offers strength and fracture toughness. The Al7475 alloy is a perfect aircraft alloy, which is suitable for use in the construction of fuselage skins, bulkheads, and wing sections for commercial fighter, and transport aircraft. Al7475 alloy provides remarkable toughness and is suitable for a variety of applications. Metal working techniques such as machining, forging, and heat treatment are some of the choices that are available with this alloy^3^. The Al7050 alloys have a very good electrical conductivity and strong corrosion-resistance features.

The high-performance ceramic substance known as zirconium carbide is well-known for its excellent qualities. Its chemical formula is ZrC. It is having high melting point approximately 3,550 °C, one of the highest among ceramics. ZrC is typically synthesized by reducing zirconium oxide with carbon at high temperatures^4^. It has high hardness, making it highly wear-resistant. It is stable at high temperatures and in various chemical environments. It has good thermal conductivity, suitable for high-temperature applications. It has uses in aerospace, the electronics industry, as well as in cutting tools and abrasives.

Liquid state, semisolid, and powder metallurgy techniques are the three production processes that are typically employed for producing particulate reinforced MMCs. Ceramic particles are added to the molten metal for producing MMCs by liquid state techniques^5^. The most essential and extensively used casting alloys are those that are made of aluminum alloys. Moreover, one reason for the increasing use of cast alloys is the possibility of enhancing their mechanical properties through heat treatment^6^.

Standard T6 heat treatment is commonly used in the manufacturing of components, which has three steps like solutionizing, quenching as well artificial ageing^7^. To evaluate how the temperature and time of solution affect the mechanical properties of aluminum alloys, many studies have been performed^8,9^. A comprehensive literature review has been performed on the microstructural study and mechanical characteristics of as-stir-cast and heat-treated stir-cast composites relied on aluminium alloy and are outlined as follows.

Krishna Mohan Singh et al. examined the wear behavior of heat-treated aluminum MMCs reinforced with B_4_C particles. The heat-treated Al7075-B_4_C composites exhibited high hardness in comparison to the Al7075-B_4_C composites that have not been heat-treated^10^. Viswanatha et al. investigated how heat treatment affected the characteristics of MMCs made from stir-cast Al-7Si alloy. The heat treatment process enabled a substantial enhancement in tensile strength of the composites^11^. Hamid M. Mahan et al. performed a study examining the influence of heat treatment on the mechanical characteristics of AA2024 aluminium alloy that was reinforced with nanoparticles. They have reported that the rapid solidification process and thermal treatment significantly improved the mechanical properties by reducing micro segregation^12^. Gurumurthy et al. studied how heat treatment affects the tensile behaviour of composites manufactured from Al7075 alloy and white cast iron. Both the Al7075 matrix and the Al7075-white cast iron composites were subjected to age hardening, resulting in a significant improvement in the mechanical properties^13^.

Raj Kumar et al. studied the Al7075 alloy composites that were reinforced with ZrO_2_ and nanographite particles. Nanographite and ZrO_2_ are introduced to significantly improve the composite’s hardness and impact strength^14^. Bharath et al. investigated the tensile behaviour of Al2014 alloy composites containing Al_2_O_3_ particle^15^. Most researchers have claimed that the mechanical properties of heat-treated Al2014-15%Al_2_O_3_ composites have improved significantly.

Xin Li et al. have evaluated the characteristics of the composite materials made of aluminium alloy and SiC. Based on the findings, it was discovered that the UTS of the heat-treated composite is much higher than that of the as-cast composite^16^.

Gopal Krishna et al. have studied the microstructure of stir-cast composite comprising of Al7075 matrix and micro WC-Co particles. The SEM micrographs showed an even dispersion of ceramic particles throughout the matrix^17^. Siddesh Matti et al. studied how heat treatment affects the mechanical performance of composites made from Al7075. According to their findings, the percentage of reinforcements and the UTS of Al7075 alloy steel composites are positively correlated, whether or not the composites have undergone heat treatment^18^. Ramadoss et al. have investigated the stir-cast composites comprising of Al7075 alloy, B_4_C and BN particulates. The results showed that adding reinforcement in Al7075 alloy led to enhance its mechanical characteristics including hardness, tensile & compression strengths^19^.

An examination of the mechanical properties of Al-ZrC-B_4_C MMCs was performed by Theerkka Tharaisanan et al. The findings have shown that addition of ZrC reinforcement has improved the hardness, UTS and impact strength^20^. Kalaiyarasan et al. have worked on the stir-cast AA8090-WC-ZrC MMCs. It was found that, the BHN of the produced composites was more than the aluminum alloy^21^.

Satyanaryana et al. have worked on the properties of stir-cast AA7020-Zirconium carbide particulate MMCs. They have reported that, the UTS of the composites have been reduced with increase in porosity and clustering of ZrC nanoparticles^22^. Satish Kumar et al. have studied the production and hardness characteristics of AZ31-ZrC composites. It was found that, the Aluminium alloy exhibited a hardness of 60 HV and Al-15 v% ZrC composite exhibited a hardness of 108 HV^23^.

Velmurugan et al. studied the mechanical behaviour of Copper-Nickle-Zirconium carbide MMCs produced utilizing stir casting. Due to the even distribution of ZrC particulates throughout the aluminium matrix, the synthesized composite exhibited substantially enhanced hardness and UTS^24^.

Although A7475 is a frequently employed aerospace-grade aluminium alloy, the majority of reinforcement studies have concentrated on the family of SiC, Al_2_O_3_, or TiC particulates. In contrast, this investigation incorporates a novel reinforcement phase into the A7475 matrix. Specifically, Zirconium Carbide (ZrC) has been identified as offering distinctive benefits, including superior thermal stability, increased hardness, and improved wear resistance. A homogeneous distribution of ZrC particles is achieved by employing a stir casting technique in. This leads to an improvement in mechanical efficiency.

Thus, these findings show that A7475-ZrC composites provide better strength and fracture toughness as contrasted to the conventional MMCs which should be the target for the next generation aerospace and automotive applications. Lightweight and stronger structural supports with improved thermal stability, the A7475-ZrC composite overlays wear-resistant brake rotors and high-performance engine components.

This study establishes a standard for aluminium matrix composites that is characterised by a balance of extraordinary strength, toughness, and wears resistance by incorporating ZrC into A7475. Contrary to previous research that primarily examined conventional reinforcements, our results establish ZrC as a reinforced phase for next-generation reinforcements and broaden the potential for high-temperature and high-stress applications.

The literature review clearly stated that, many researchers have worked on the area of aluminum based particulate reinforced composites; however, no extensive work has been performed on the Al7475-ZrC composites. The impact of T6 heat treatment on Al7475-ZrC MMCs has not been previously investigated. The present study focused on the fabrication of Al7475-ZrC composites and evaluation of the heat treatment effect on the properties of Al7475-ZrC MMCs. The resulting composites are employed in automotive components, such as brake drums, pistons and connecting rods. Certain automobile parts including connecting rods and pistons in IC engines are subjected to extreme temperature. Hence they will be subjected to wear and tear and their strength decreases. Since Al7475 alloy and Zirconium carbide particles can sustain high temperatures, the composites made from Al7475 and ZrC particles has the capacity to withstand high temperatures. Hence in the present work Al7475-ZrC composite has been subjected to T6 heat treatment.

## Materials and methods

Table 1 indicates the constituents of Al7475 alloy.

**Table 1:** Constituents of Al7475 alloy.

Element	Aluminium	Zinc	Magnesium	Copper	Chromium	Iron	Silicon	Manganese	Titanium
Quantity (wt%)	Balance	5.5	2.2	1.4	0.2	0.1	0.08	0.04	0.04

**Fig. 1:**
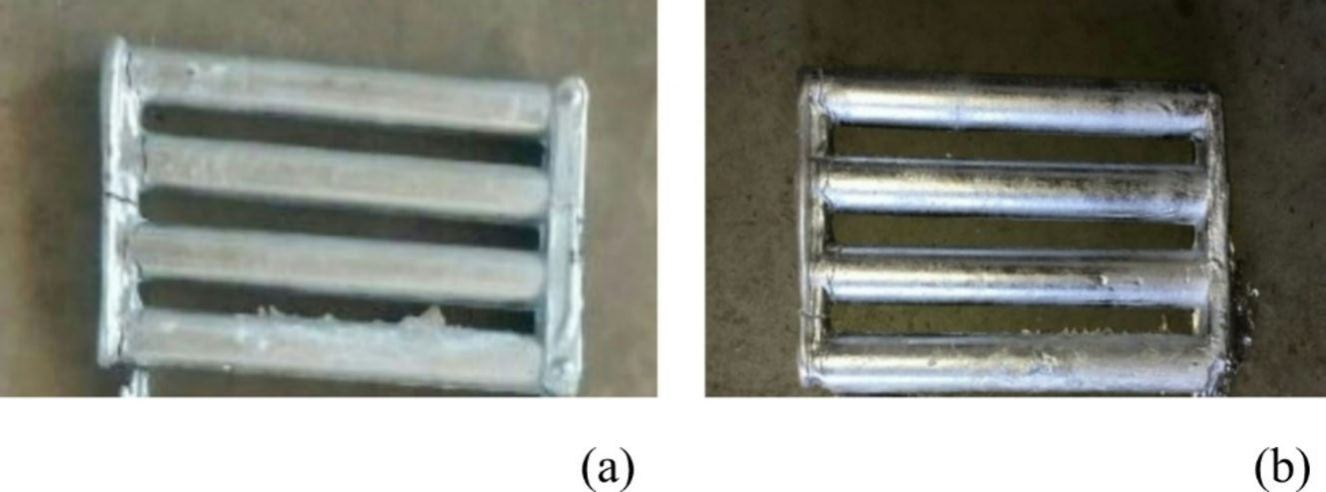
As-stir-cast and heat-treated stir-cast Al7475-6%ZrC composite samples.

Al7075-ZrC composites were processed by stir casting technique. The Al7475 alloy was melted in a furnace for 700 °C, and then mechanically stirred vigorously with a stainless steel mechanical stirrer at 300 rpm to create a vortex. To this melt, a 2 wt% of ZrC particles which were preheated to 400 °C was added slowly under continuous stirring for 10 min. The molten metal is then put into a mould that has been preheated to 300 degrees Celsius, and the mould is then allowed to cool down^27^. The process was repeated for producing composites with 4, 6 and 8 wt% of ZrC particles. Figures 1a and b depict the as-stir-cast and heat-treated stir-cast Al7475-6%ZrC composite samples.

A muffle furnace was used to perform a T6 heat treatment on the Al7475 alloy and Al7475-ZrC composites. A solution heat treatment was performed for two hours at 530 degrees celsius. After undergoing solution heat treatment, the samples were naturally aged at 175 degrees celsius for a period of six hours before being brought down to room temperature in air^28^.

Standard metallographic procedure was used for producing the Al7075-ZrC composite specimens of 10 millimetres by 5 millimetres in size. Keller’s reagent was subsequently employed to etch the specimens. SEM device was utilized to invrstiagte the microstructure of the composites. According to ASTM-E-10-93 standard, the Brinell hardness tester was used to perform the measurement of the composite’s hardness. The force used was 500 kilogrammes, and the dwell time was two seconds. The tensile tests were conducted using the universal testing apparatus following the ASTM E8M-09 standard, while the compression strength tests were carried out on a universal testing apparatus with a capacity of ten tonnes, adhering to the ASTM B577 standard. The impact tests were conducted on the composite samples of size 55 mm x 6 mm in an impact testing machine as per ASTM A370 standard.

Equation (1) is utilized to estimate the hardness of Al7075-ZrC composites.

1 $$\:\text{B}\text{r}\text{i}\text{n}\text{e}\text{l}\text{l}\:\text{H}\text{a}\text{r}\text{d}\text{n}\text{e}\text{s}\text{s}\:\text{N}\text{u}\text{m}\text{b}\text{e}\text{r}=\frac{2xP}{\pi\:xD\:[D-\sqrt{{D}^{2}-{d}^{2}}]}$$

Where,

P = Load (kg),

D = Indenter diameter (mm) and.

d = Indentation diameter (mm).

## Results and discussion

### EDX study of Al7475 alloy & Al7475-ZrC composite

**Fig. 2:**
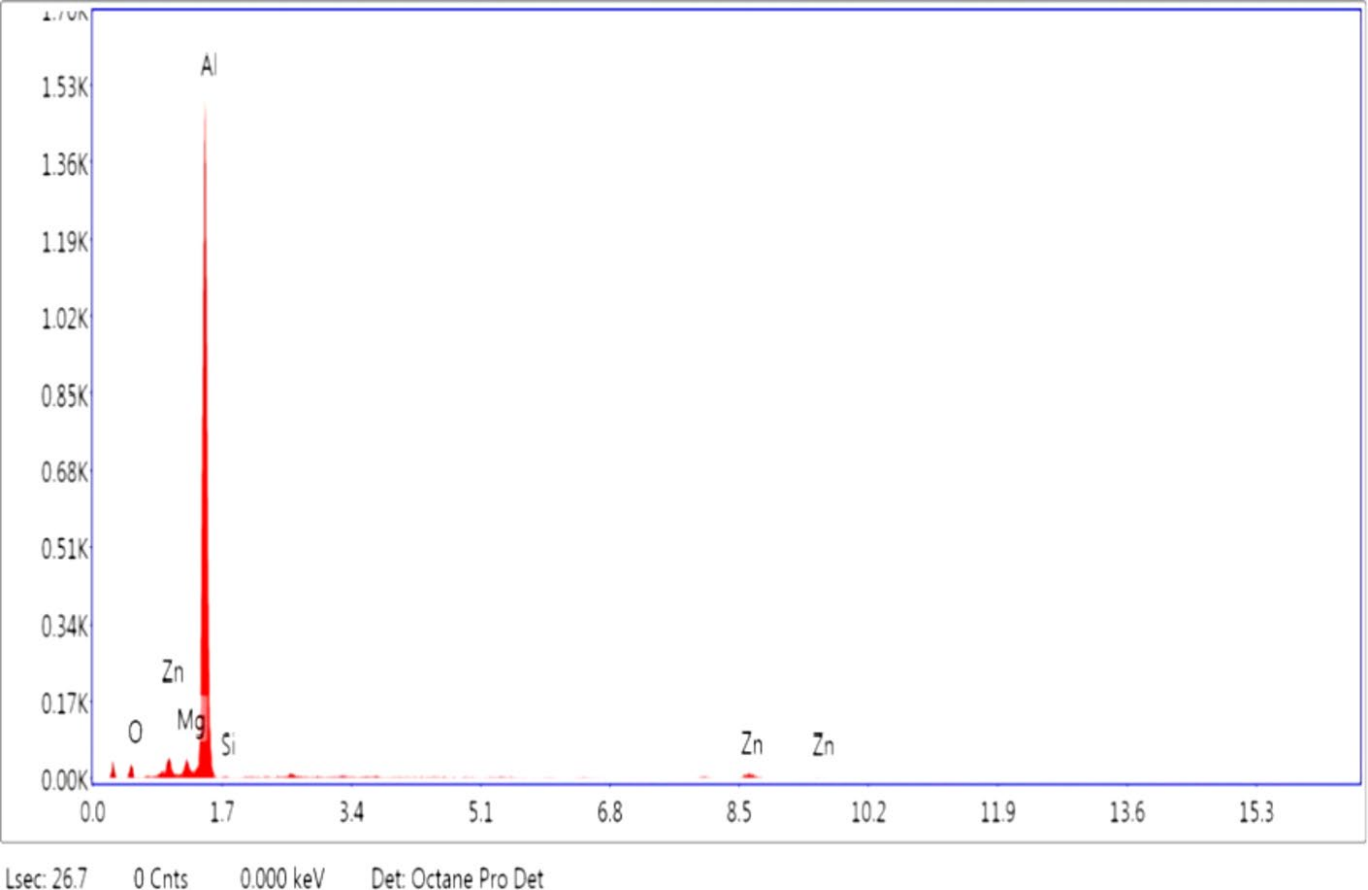
EDX spectrum of Al7475 alloy.

Figure 2 presents the EDX image of Al7475 aluminium alloy. It shows the presence of Aluminium, Zinc, Magnesium and Silicon.

**Fig. 3:**
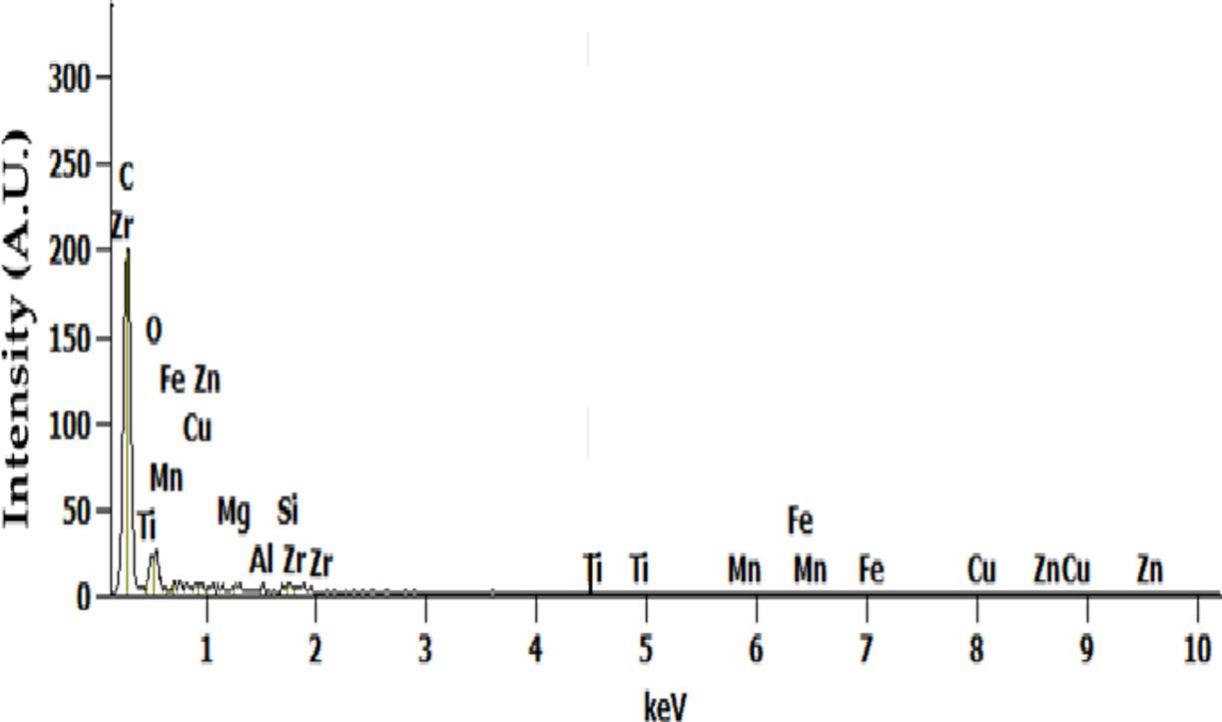
EDX spectrum of as-stir-cast Al7475-6%ZrC composite.

The EDX spectrum of as-stir-cast Al7475-6%ZrC composite is shown in Fig. 3. It shows the presence of Aluminium, Zirconium and Carbon and traces of other elements. Elemental mapping using EDS can further confirm the homogeneous distribution of ZrC particles.

### SEM analysis of aluminum Al7475 alloy and Al7475-ZrC composites in as-stir-cast condition

**Fig. 4:**
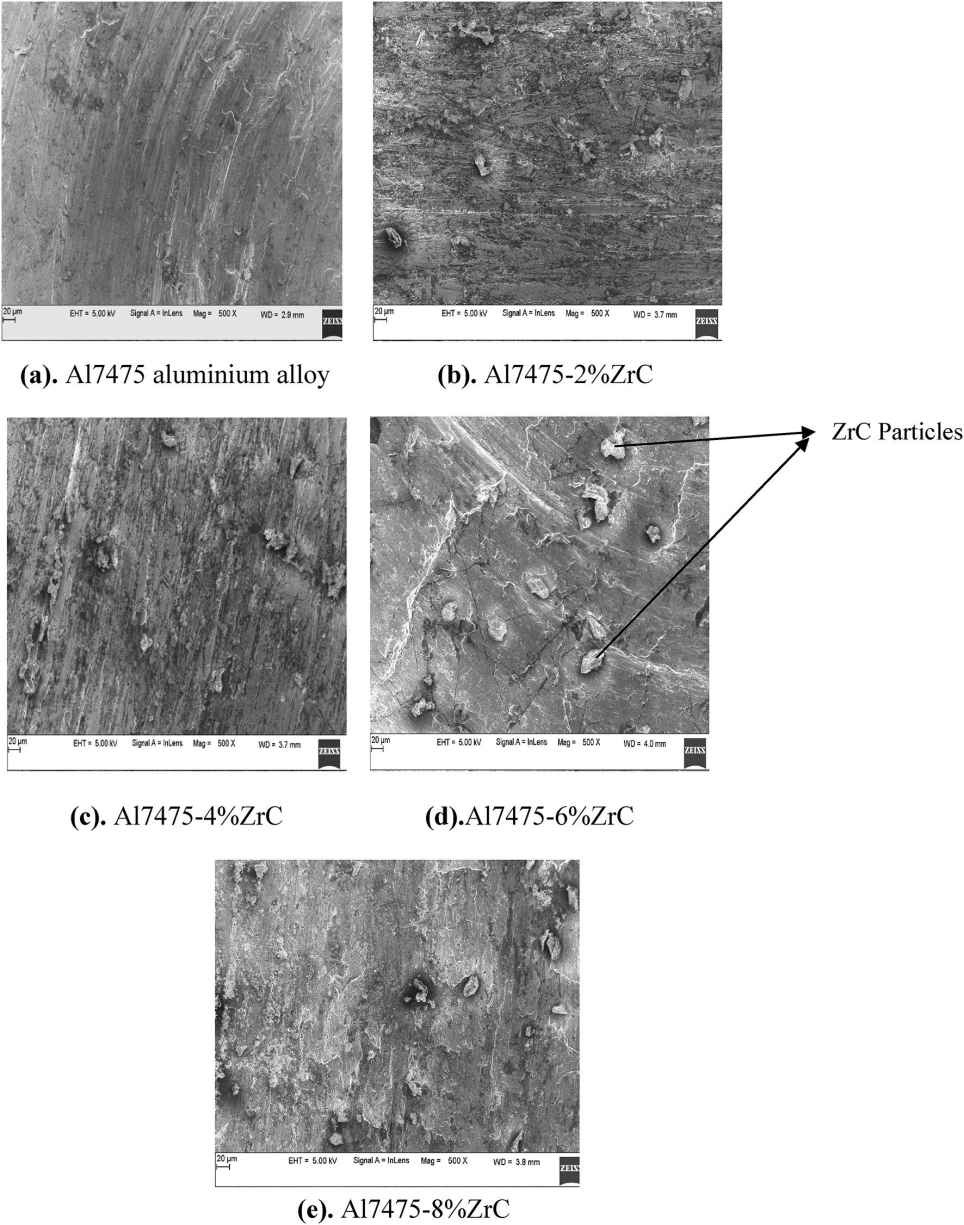
SEM micrographs of as-stir-cast Al7475 matrix and Al7475-ZrC composites.

The SEM micrographs of Al7475 aluminium alloy and Al7475-2%ZrC, Al7475-4%ZrC, Al7475-6%ZrC and Al7475-8%ZrC composites in as-stir-cast condition are shown in Figs. 4(a) to 4(e). Figures 4(b) to 4(d) demonstrate that ZrC particles are equally dispersed inside the Al7475 matrix. The ZrC particles are consistently distributed in the Al7475 matrix owing to preheated mould and constant agitation during the melting of the mixture^25^. Micrographs obtained using SEM show that the quantity of ZrC particulates that are distributed in the matrix of Al7475 increases as the number of ZrC particulates in the matrix increases.

The microstructure-mechanical property correlation for Al7475-ZrC AMCs indicate a uniform distribution of ZrC with rigid interfacial adhesion, which leads to a significant enhancement of strength, hardness, and wear resistance^26^.

Uniformly distributed ZrC particles enhanced mechanical and wear properties via grain refining and dislocation pinning. During solidification, the ZrC particles serve as a nucleation site in solidification refining the Al7475 matrix thereby improving mechanical properties of the composites. A strong bond between the Al7475 and ZrC can minimize areas of weakness, which leads to a lower chance of failure and improves the mechanical properties of the composites. Pores or agglomerated ZrC particles can lead to worsening mechanical performance^27^.

The harder and more rigid ZrC particles take part of the load which helps alleviate stress on the softer Al7475 matrix. This mechanism improves UTS as well as yield strength. The ZrC particulates contribute to the process of preventing the expansion of the grain throughout the solidification and processing procedure. The motion of dislocations can be impeded by the reduction in the size of the grains that comprise the animated microstructure and the increase in the area of the grain borders. This improves both hardness and strength of the material^28^. Addition of ZrC particles helps in increasing the dislocation generation at matrix-reinforcement interface. The greater energy needed to move dislocations increases strength and hardness.

The SEM images of Al7475-ZrC composites show that ZrC particulates are evenly dispersed in the Al7475 matrix indicating good mixing and dispersion method. The ZrC particles are not agglomerated in the SEM images, leading to enhanced mechanical properties. The SEM images indicate good interfacial bonding among the Al7475 matrix and ZrC particulates. The outcome is enhanced mechanical functionality. The absence of interfacial voids in the SEM images leads to an improved composite performance^29,30^. The SEM images also show the formation of reaction products like Al_3​_Zr at the interface. The finer grains can be viewed in the scanning electron pictures with the inclusion of ZrC particulates which improved the strength of the composites. The SEM images confirm the absence of porosity in the composite, resulting in improved mechanical properties. It is well-observed from the SEM images a good distribution, good interfacial adherence and low porosity. It results in excellent mechanical and wear properties^31^.

**Fig. 5:**
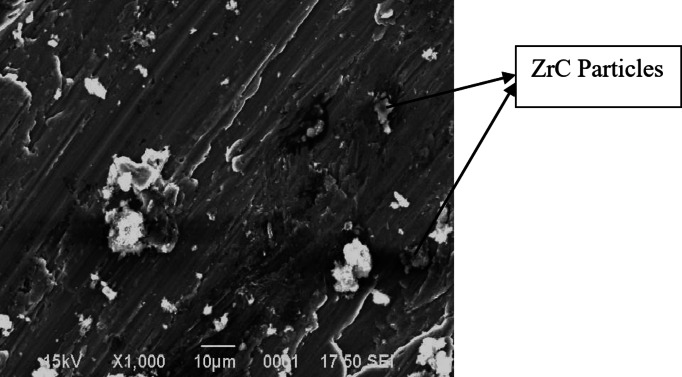
SEM picture of Al7075-6%ZrC composite at 1000X magnification.

The high magnification SEM images of Al7475-ZrC composite more clearly ensure that, ZrC particulates are homogenously dispersed in the aluminium matrix. Porosity and voids are seen clearly at high magnification. Figure 5 indicates the SEM picture of Al7075-6%ZrC composite at 1000X magnification.

### SEM analysis of T6 heat-treated stir-cast Al7475 alloy and Al7475-ZrC composites

**Fig. 6:**
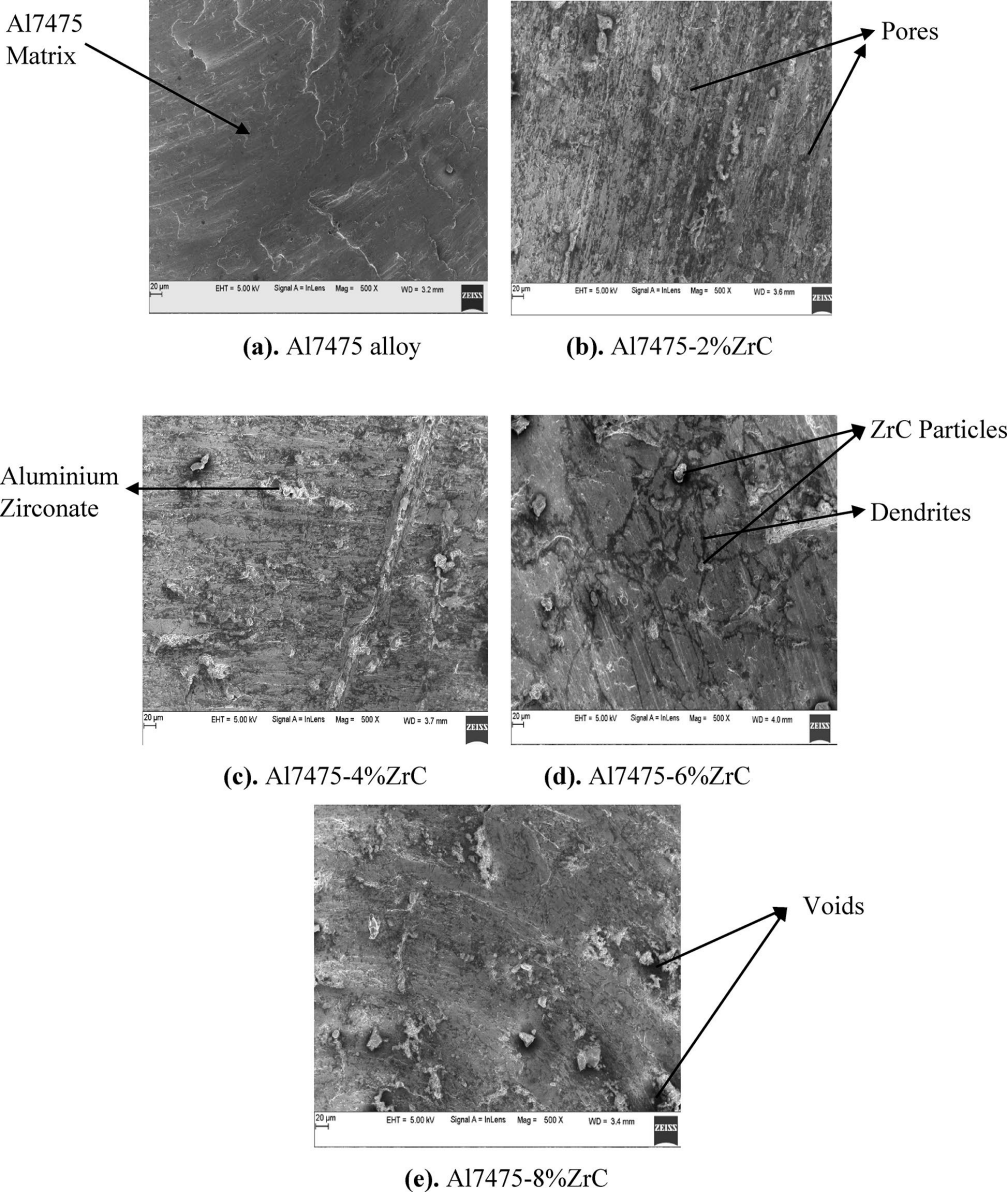
SEM micrographs of heat-treated stir-cast Al7475 alloy and Al7475-ZrC composites.

The SEM micrographs of heat-treated stir-cast Al7475 alloy, Al7475-2%ZrC, Al7475-4%ZrC, Al7475-6%ZrC and Al7475-8%ZrC composites are depicted in Figs. 6(a) to 6(e). The ZrC particles have been observed to be equally dispersed throughout the aluminium matrix, as depicted in Figs. 6(b) to 6(d). As illustrated in Fig. 5, the amount of ZrC particles within the Al7475 matrix is increased in composites with higher levels of ZrC particulates. Ramadoss et al. also reported similar findings. The findings demonstrated that, the ZrC particulates are distributed consistently throughout the Al7075 matrix. As a result of the heat treatment, the microstructures of composites reveal that the grain has been refined. Consequently, mechanical characteristics of the composites are enhanced.

### Tensile strength of Al7475 alloy and Al7475-ZrC composites in as-stir-cast and heat-treated stir-cast condition

**Fig. 7:**
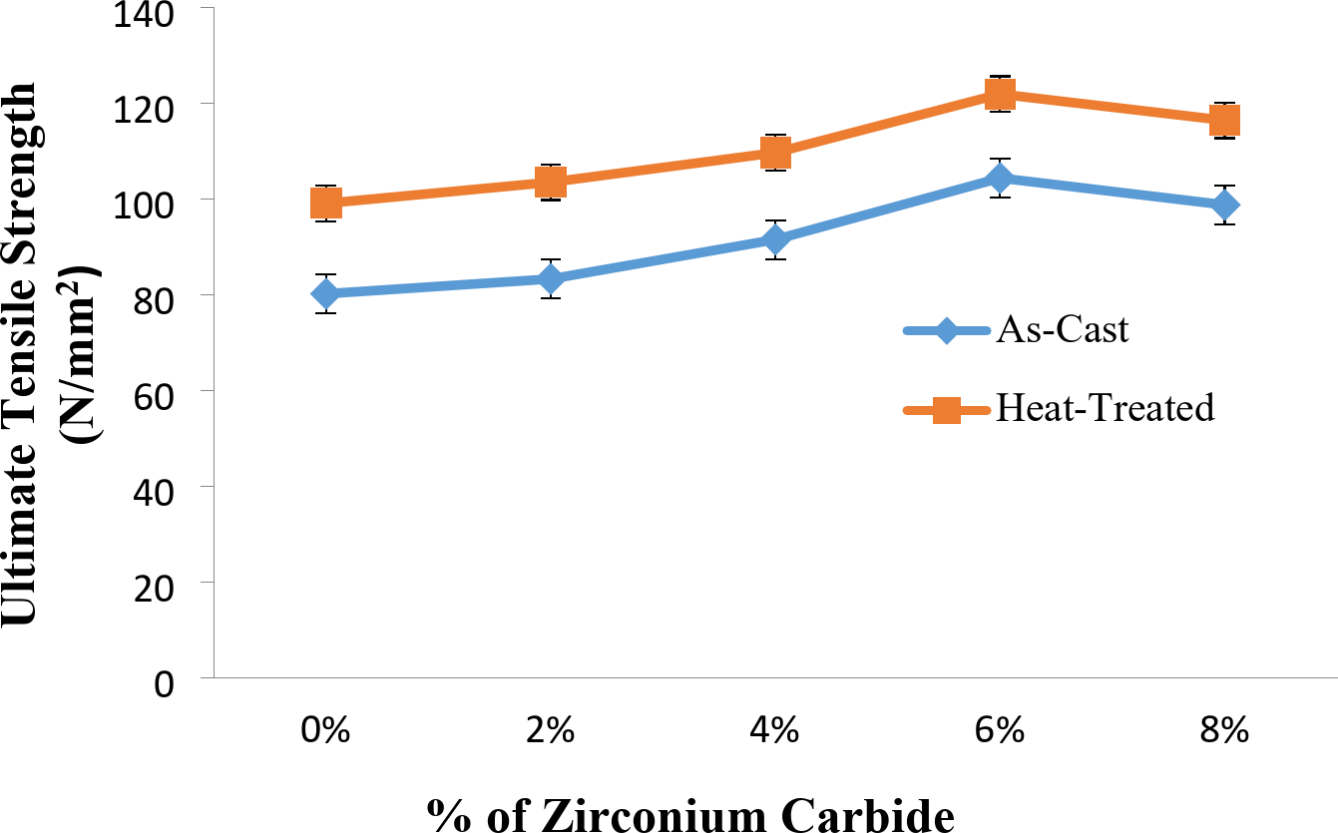
UTS vs. % of ZrC of as-stir-cast and heat-treated stir-cast Al7475-ZrC composites.

The graph of UTS vs. % of ZrC of heat-treated stir-cast Al7475 alloy and Al7475-ZrC MMCs is presented in Fig. 7. The above figure shows that, the UTS improved up to 6% of ZrC, and then it is decreased. Similarly, for compression, hardness and impact tests, the values of compression strength, hardness and impact strength were optimum at 6% of ZrC and then it is decreased. This is due to the fact that, Al7475 alloy and ZrC particles have a low moisture absorption capacity and also the agglomeration of ZrC that makes the ZrC particles attach together. This eventually reduces strength of the composites. Zirconium carbide particles act as nucleation sites and help to get finer grains during solidification^32^. Increased yield strength is noted with finer grain size, as per the Hall-Petch relationship, which indicates that yield strength rises with reduced grain size. The improvement in strength is a result of successful stress transmission from the comparatively soft matrix material to the relatively hard reinforcement particles^33^. This can be due to the stronger interfacial bonding that exists among the ZrC particulates and the aluminium matrix. The ZrC particles are uniformly disseminated throughout the aluminium matrix. These ZrC particles serve as an impediment for the dislocation motion. This leads to rise in composite’s hardness and strength. Thus, these particles play the role of obstacles and stress is needed in excess of these to cause plastic deformation. The ZrC particles hinder dislocation motion by forcing dislocations to bow around them which increase the strength via the Orowan mechanism. The thermal mismatch and CTE discrepancy between the matrix and reinforcement are further exacerbated by the ZrC particulates, resulting in the growth of dislocations^34^. As a result, the density of dislocation increases, which enhances the hardness and strength. The presence of ZrC particles provides remarkable thermal stability and restricts the extent of grain growth when the sintered materials were exposed to elevated temperature resulting in the retention of the mechanical integrity of the composite^33^. However, beyond 6 wt% of ZrC particles, the composite’s mechanical properties degraded significantly due to particle agglomeration, porosity formation, and reduced wettability between the matrix and reinforcement. As a result, this negatively affected the load transfer efficiency and the overall strength of the produced composite.

Wettability is directly linked to interfacial bonding, mechanical properties and composite performance. Poor wetting often results in interfacial voids and weak bonding, consequently detrimental to load transfer, while good wettability can improve adhesion and mechanical interconnectivity. Mechanical properties are indirectly affected by wettability, including tensile strength and hardness. Interfacial analysis via SEM can be used to analyze wettability^35^.

Indirect confirmation of wettability is achieved through the identification of interfacial voids or reaction layers in SEM images. EDS mapping apprehends diffusion and reaction products such as Al_3_​Zr that will affect the adhesion. Both observations of particle debonding and fracture mode indicate wettability. With good wettability, the matrix will fail in a cohesive manner, while poor wettability will lead to debonding of the matrix and fiber interface.

In T6 heat-treated stir-cast Al7475-ZrC composites, the highest tensile strength is obtained at 6% ZrC particles. The UTS of heat-treated stir-cast Al7475-ZrC MMCs are higher than that of as-stir-cast composites. The reason for this is the development of fine grains and precipitates as an outcome of the heat treatment. The highest UTS for as-stir-cast Al7475-6%ZrC composite is 104 N/mm^2^, while it is 122 N/mm^2^ for T6 heat-treated stir-cast Al7475-6%ZrC composite. A 15% increase in tensile strength is attained compared to as-stir-cast Al7475-6%ZrC composite. Similar outcomes were attained by Gurumurthy et al.^13^. The heat-treated stir-cast Al7075-white cast iron composites exhibited high tensile strength in comparison to the Al7075-white cast iron composites that have not been heat-treated. The UTS values of Al7075-White cast iron composites were enhanced by 17% after heat treatment.

#### Tensile fracture analysis of as-stir casted Al7475 alloy and Al7475-ZrC composites

**Fig. 8:**
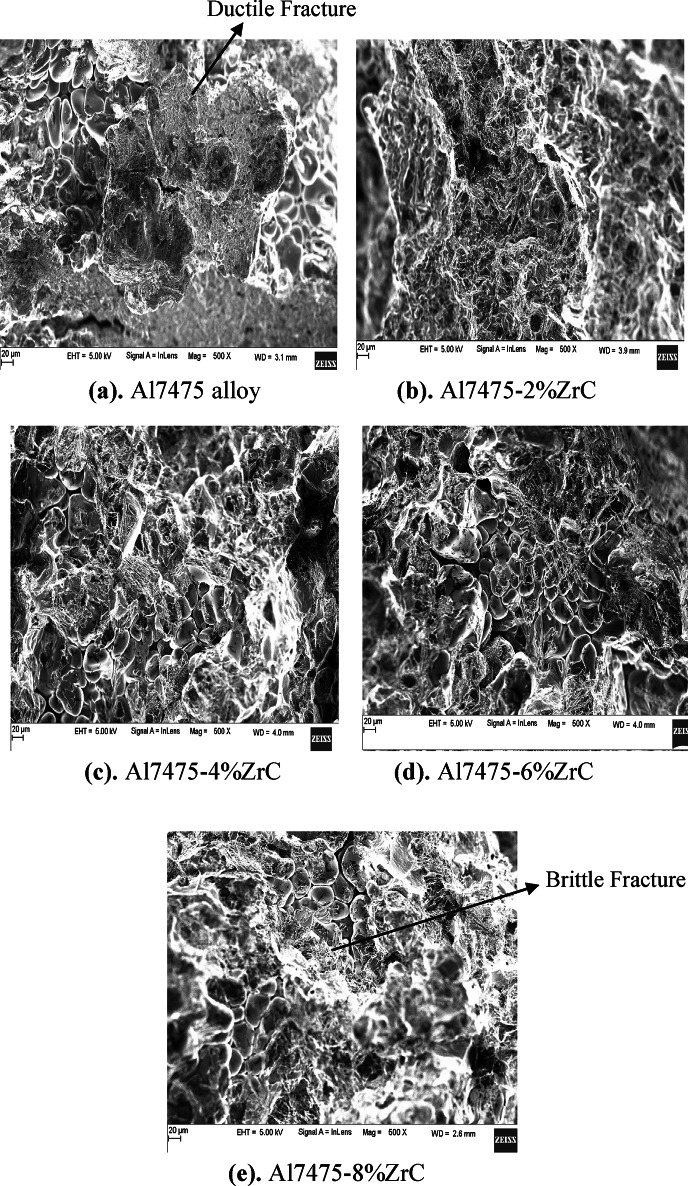
As-stir-cast Al7475 alloy and Al7475-ZrC composites tensile fractured surfaces.

Figures 8(a) to 8(e) depict the tensile fracture surfaces of as-stir-cast Al7475 alloy & Al7475-2%ZrC, Al7475-4%ZrC, Al7475-6%ZrC and Al7475-8%ZrC composites respectively. Tensile fracture surface of Al7475 alloy as shown in Fig. 8(a) reveals ductile fracture nature of Al7475 matrix. From the tensile fractured surfaces presented in Figs. 8(b) to 8(d), it is apparent that, the number of cracked ZrC particles increases with the increment of ZrC particulate content in the Al7475 matrix. The hardness of the composite increases with the number of the fractured zirconium carbide particles in the Al7475 matrix. As the amount of ZrC particles in the Al7475 matrix increases, the composite material’s ductility diminishes. The brittleness of the composites gets increased due to which their strength is also increased. The strength of Al7475 matrix is also one more factor in the enhancement of composite’s strength. The improved composite’s strength is owing to the wettability among the aluminium matrix and zirconium carbide particles. Figures 8(b) to 8(d) show the tensile fracture surfaces, demonstrating the ZrC particles adhesion to the Al7475 matrix. This indicates ZrC particles fractures first followed by Al7475 matrix fracture. The propagation of cracks in the Al7475 matrix is impeded by ZrC particles, which act as an obstruction. Because of this, the developed composite has more strength than the Al7475 alloy. The scanning electron picture of the Al7475-2%ZrC composite fractured surface show a lower percentage of ZrC particles in the Al7475 matrix compared to the Al7475-6%ZrC composite. The composite’s strength and resilience are influenced by the zirconium carbide particles within the Al7475 matrix. There are more clusters of ZrC particles inside the given region of the fracture surface of the Al7475-6%ZrC composite, which results in improving the composite’s strength. In this particular instance, the particles are bearing a greater load than the matrix^36^. As a result of the tensile test, it was discovered that the Al7475-6%ZrC composite strength is higher than the Al7475 matrix. It is possible that the existence of ZrC reinforcing particles might be responsible for the brittleness that is found in composites.

It has been found that broken ZrC particles substantially assist in dispersion strengthening and inhibit dislocation motion. This enhances the composite’s strength. Conversely, increasing the amounts of ZrC in the solution causes the material to become brittle as hard ceramic phases dominate, adversely affecting its ductility and fracture toughness^37^.

Increasing ZrC content improves hardness as well as composite’s strength as it employs several reinforcement mechanisms such as transfer of load and refinement of grain. Fragmentation of ZrC particles during mechanical loading also serves as a mechanism for further strengthening of the composites since particle fragments can provide additional sites for matrix reinforcement. These fragmented pieces help in dispersion strengthening acting as dislocation pinning sites, preventing dislocations from moving, thus improving hardness and strength.

#### Tensile fracture analysis of heat-treated stir-cast Al7475 alloy and Al7475-ZrC composites

**Fig. 9:**
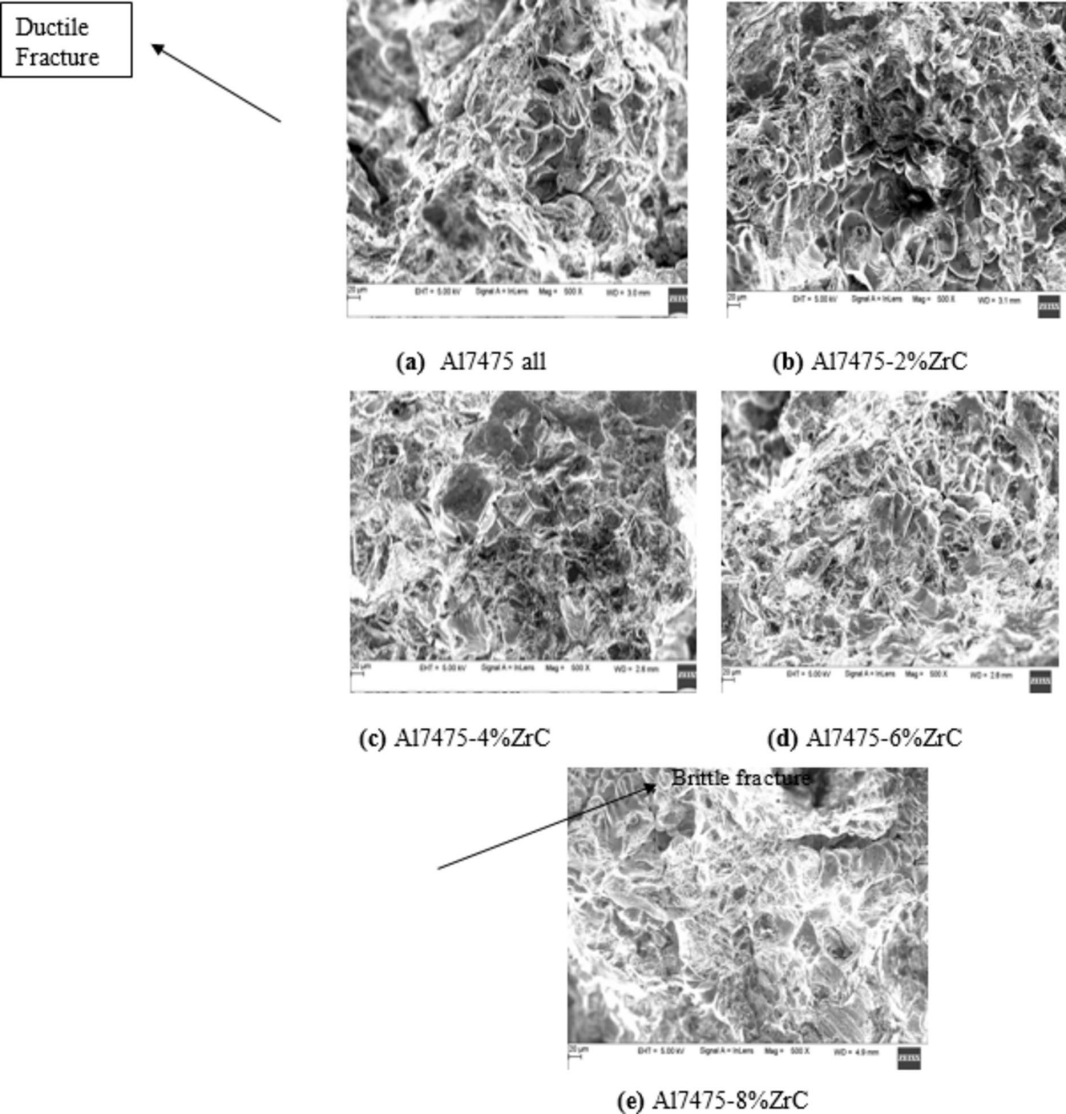
Tensile fractured surfaces of heat-treated stir-casted Al7475 alloy and Al7475-ZrC composite.

The SEM fractographs of heat-treated Al7475 alloy, Al7475-2%ZrC, Al7475-4%ZrC, Al7475-6%ZrC and Al7475-8%ZrC composites are depicted in the Figs. 9(a) to 9(e). It is more likely that, the grain refinement will be high in composites as a result of heat treatment. Because of this, the composites become more brittle but the strength of the heat-treated stir-cast Al7475-ZrC composite will be increased due to grain refinement.

As seen in Figs. 9(b)-9(d), with the increase of ZrC concentration in the Al7475 matrix, the fractured ZrC particles increases^38^. An increase in the number of fractured ZrC particulates in the Al7475 matrix results in an improvement in the composite’s strength. However, the brittleness will increase as the content of ZrC particles in the Al7475 matrix is proportionally increased. Thus the composite’s strength will also be improved. The tensile fractured surfaces reveal that the ZrC particulates were well reinforced in the Al7475 matrix as depicted in Figs. 9(b) to 9(d). The ZrC particles provide reinforcement for the Al7475 matrix, exerting resistance to crack propagation in the forefront of the fracture. Additionally, this demonstrates that the composites have a greater strength rate compared to the Al7475 alloy. The SEM fractograph of Al7475-2%ZrC composite also consisted of a lower fraction of ZrC particles in the Al7475 matrix as compared to that of the Al7475-6%ZrC composite. ZrC particulates present in the Al7475 matrix showed resistance to composite failure^39^. The fractured surface of the Al7475-6%ZrC composite has a greater number of clusters of ZrC particles within the specified region, which yields a higher strength in the composite. The ZrC particles carry more load than the matrix. From the tensile test, it is found that Al7475-6%ZrC composite gives better strength than the Al7475 alloy. In case of composite the reinforcement particles produce brittleness at 8% of ZrC.

### Yield strength of as-stir-casted and heat-treated stir-cast Al7475 alloy and Al7475-Zirconium carbide composites

**Fig. 10:**
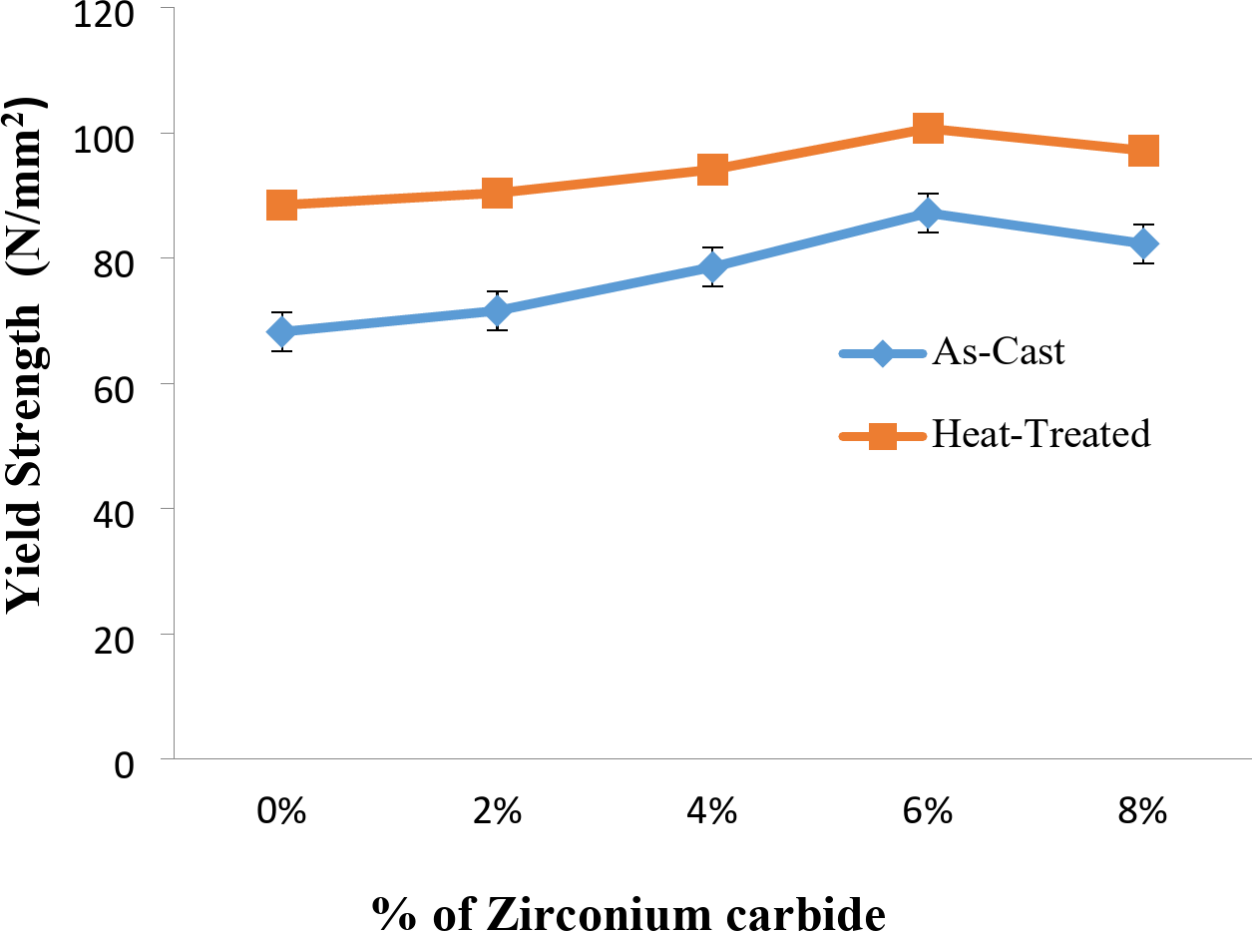
Yield Strength vs. % of ZrC of as-stir-cast and heat-treated stir-cast Al7475-ZrC composites.

Figure 10 depicts the variation of yield strength of as-stir-cast and heat-treated stir-cast Al7475-ZrC composites. Increasing the % of ZrC particles increases the yield strength up to 6% of ZrC, beyond that it decreases due to the agglomeration of reinforcement particles^40^. The yield strength is high for both as-stir-cast and heat-treated stir-cast Al7475-6%ZrC MMCs. The yield strength of heat-treated stir-cast Al7475-ZrC composites is greater than that of as-stir-cast composites. This is due to fine grain refinement and formation of fine precipitates.

It has been observed that the maximum yield strength is 87 N/mm^2^ for as-stir-cast Al7475-6%ZrC composite and 101 N/mm^2^ for T6 heat-treated stir-cast Al7475-6%ZrC composite. The yield strength of heat-treated stir-cast composite is 14% more than the as-stir-cast composite.

### Percent elongation of Al7475 alloy and Al7475-ZrC composites before and after heat treatment

**Fig. 11:**
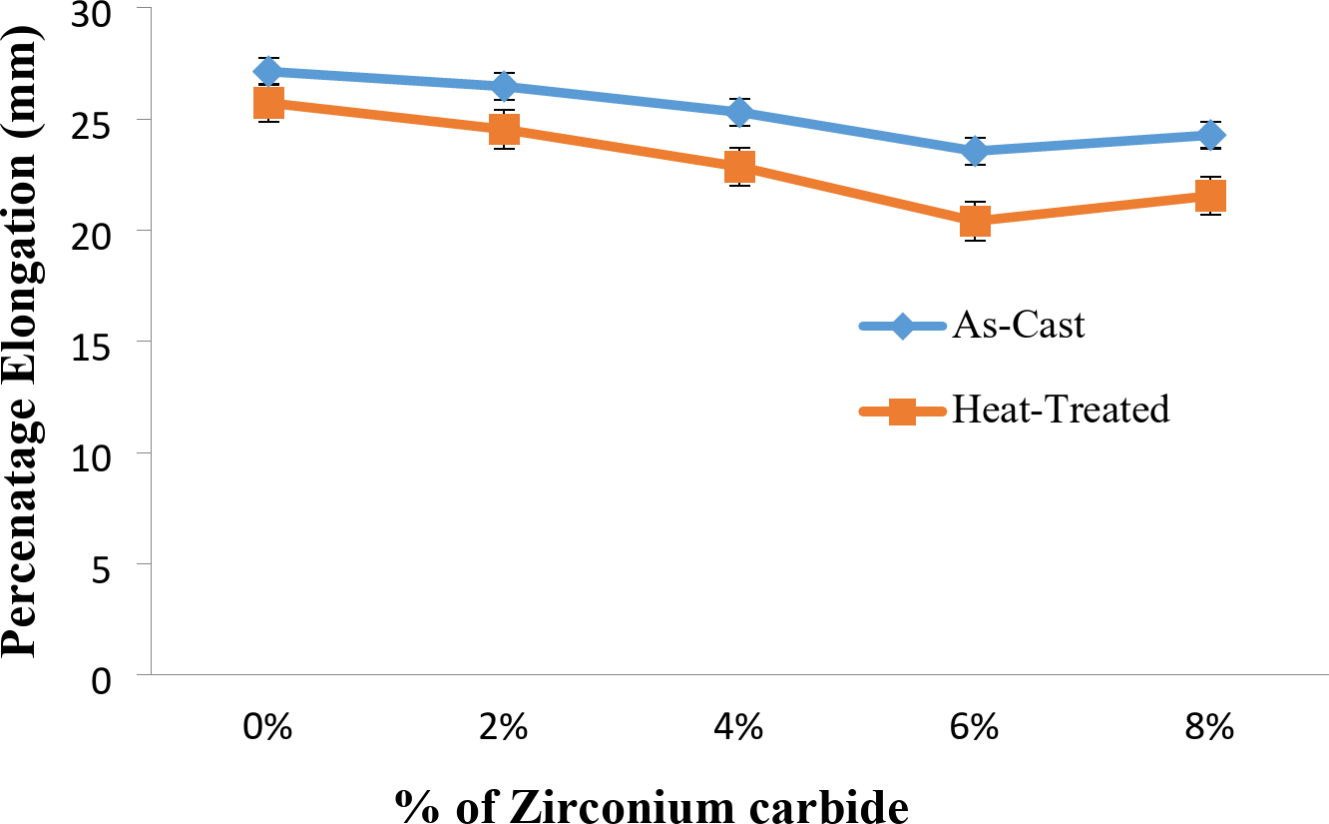
Percenatge elongation vs. % of ZrC of as-stir-cast and heat-treated stir-cast Al7475-ZrC composites.

Figure 11 illustrates the variation in percent elongation with % of ZrC particles of as-stir-cast and heat treated Al7475-ZrC composites. Figure 10 reveals that, the percent elongation is high for as-casted and heat-treated stir-cast Al7475-6%ZrC composites. The % elongation of heat-treated stir-cast Al7475-ZrC composites are less than that of as-stir-cast composites^38^. The fracture of Al7475 alloy exhibits ductility due to the absence of zirconium carbide particulates. The hardness of ZrC particles exceeds that of the Al7475 matrix. Composite strength is increased by adding hard ZrC particles to the Al7475 matrix. Adding zirconium carbide particles to Al7475 alloy decreases the matrix’s grain size and increases reinforcement-matrix adhesion. The augmentation of bonding enhances the composite’s strength while diminishing its flexibility^41^. For as-stir-cast Al7475-6%ZrC composites, the maximum % elongation is 24 mm, while for T6 heat-treated stir-cast Al7475-6%ZrC composite, maximum % elongation is 20 mm. A 17% decrease in percent elongation could be observed in heat-treated stir-cast composites when contrasted with the as-stir-cast composites.

### Compression strength of as-stir-cast and heat treated stir-cast Al7475 alloy and Al7475-ZrC composites

**Fig. 12:**
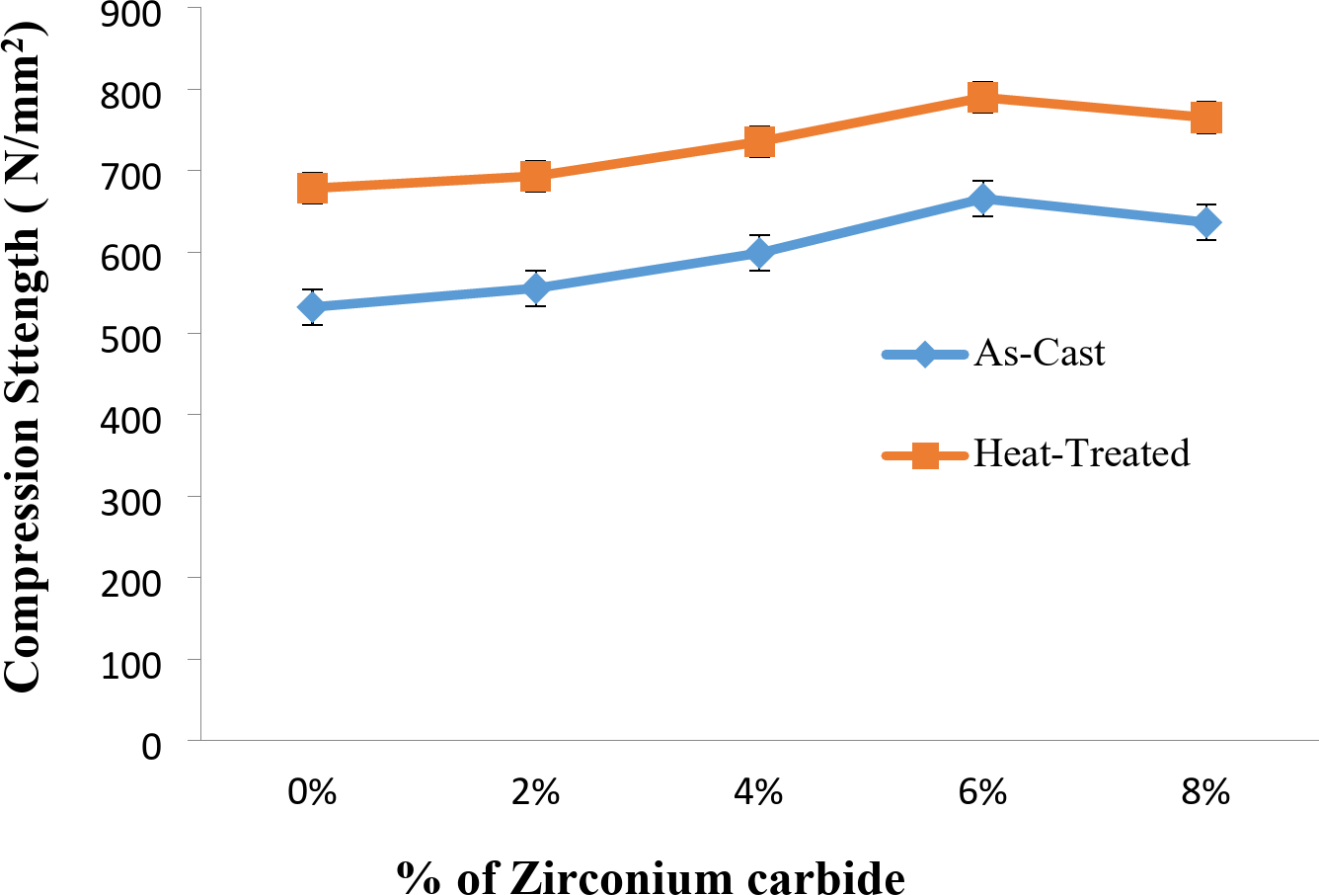
Compression strength vs. % of ZrC of as-stir-cast and heat-treated stir-cast Al7475-ZrC composites.

Figure 12 illustrates the variation in compression strength with % of ZrC particles of as-stir-casted and heat-treated stir-cast Al7475-ZrC composites. From the Fig. 11, it has been noticed that the compression strength is high for as-stir-casted and heat-treated stir-cast Al7475-6%ZrC composites. The compressive strength of heat-treated stir-cast Al7475-ZrC composites is higher than that of as-stir-casted composites^42^. This is again due to refinement of microstructure during the heat treatment. Similar outcomes were attained by Gopal Krishna et al.^17^. The heat-treated stir-cast Al7075 alloy-Nanographite-ZrO_2_ particles composites exhibited high compression strength in comparison to the Al7075-Nanographite-ZrO_2_ composites that have not been heat-treated. The compression strength values Al7075-Nanographite-ZrO_2_ composites were enhanced by 18% after heat treatment.

The highest compression strength is 665 N/mm^2^ for as-stir-cast Al7475-6%ZrC composite, and 790 N/mm^2^ for heat-treated stir-cast Al7475-6%ZrC composite. The compression strength has been improved by 16% for T6 heat-treated stir-cast Al7475-6%ZrC composite compared to as-stir-cast composite.

### Hardness of Al7475 alloy and Al7475-ZrC composites before and after heat treatment

**Fig. 13:**
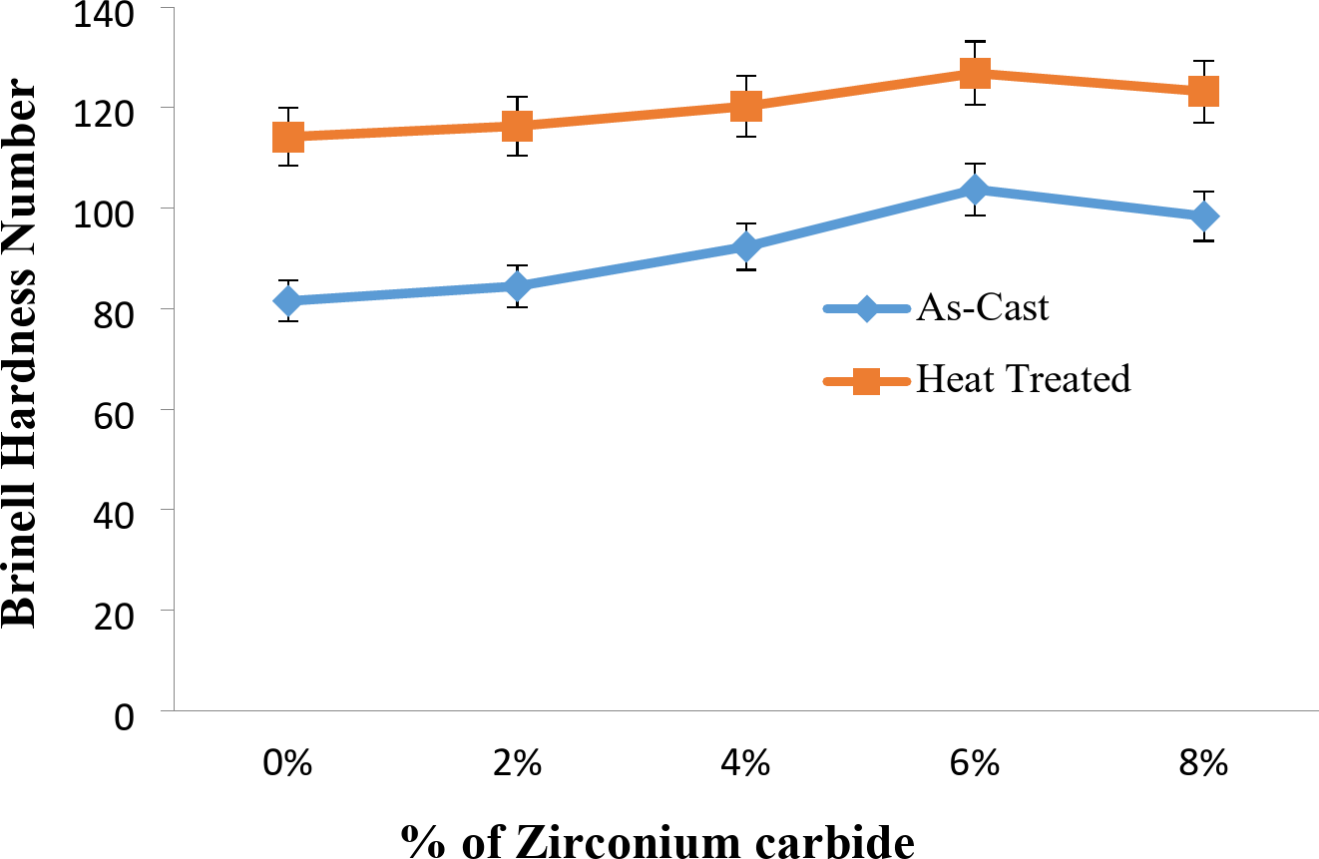
Variation of hardness with % of ZrC of as-stir-cast and heat-treated stir-cast Al7475-ZrC composites.

Figure 13 illustrates the graph of hardness vs. % of ZrC of as-stir-casted and heat-treated stir-cast Al7475-ZrC MMCs. The Fig. 12 demonstrates that the hardness improves up to 6% of ZrC, particles and then it begins to decline beyond 6% of ZrC^43^. This is due to the agglomeration of ZrC particles that decreases the bonding between the Al7475 matrix and ZrC particles. For T6 heat-treated stir-cast Al7475-ZrC composites, the hardness is at its highest when the ZrC is 6%. Similar behavior was observed by R. Vijayakumar^39^ et al. The studies found the optimal hardness at 6% of ZrC is 101 BHN for heat-treated stir-cast Al7475-ZrC composite. The hardness values of heat-treated stir-cast Al7475-ZrC composites are greater than for the as-stir-cast composites. For Al7475-6% composite, the maximum hardness obtained was 104 BHN for as-stir-cast composites and 127 BHN for T6 heat-treated stir-cast composites. Heat-treated composite is found to be 18% harder than that of as-stir-cast composite^44,45^. Krishna Mohan Singh et al. achieved similar results. The heat treated stir-cast Al7075-B4C composites had a higher hardness than the as-stir-cast Al7075-Boron carbide composites. A heat treatment resulted in an increase of around 20% in the BHN values of Al7075-B4C composites.

### Impact strength of Al7475 alloy and Al7475-ZrC in as-stir-cast and heat-treated stir-cast condition

**Fig. 14:**
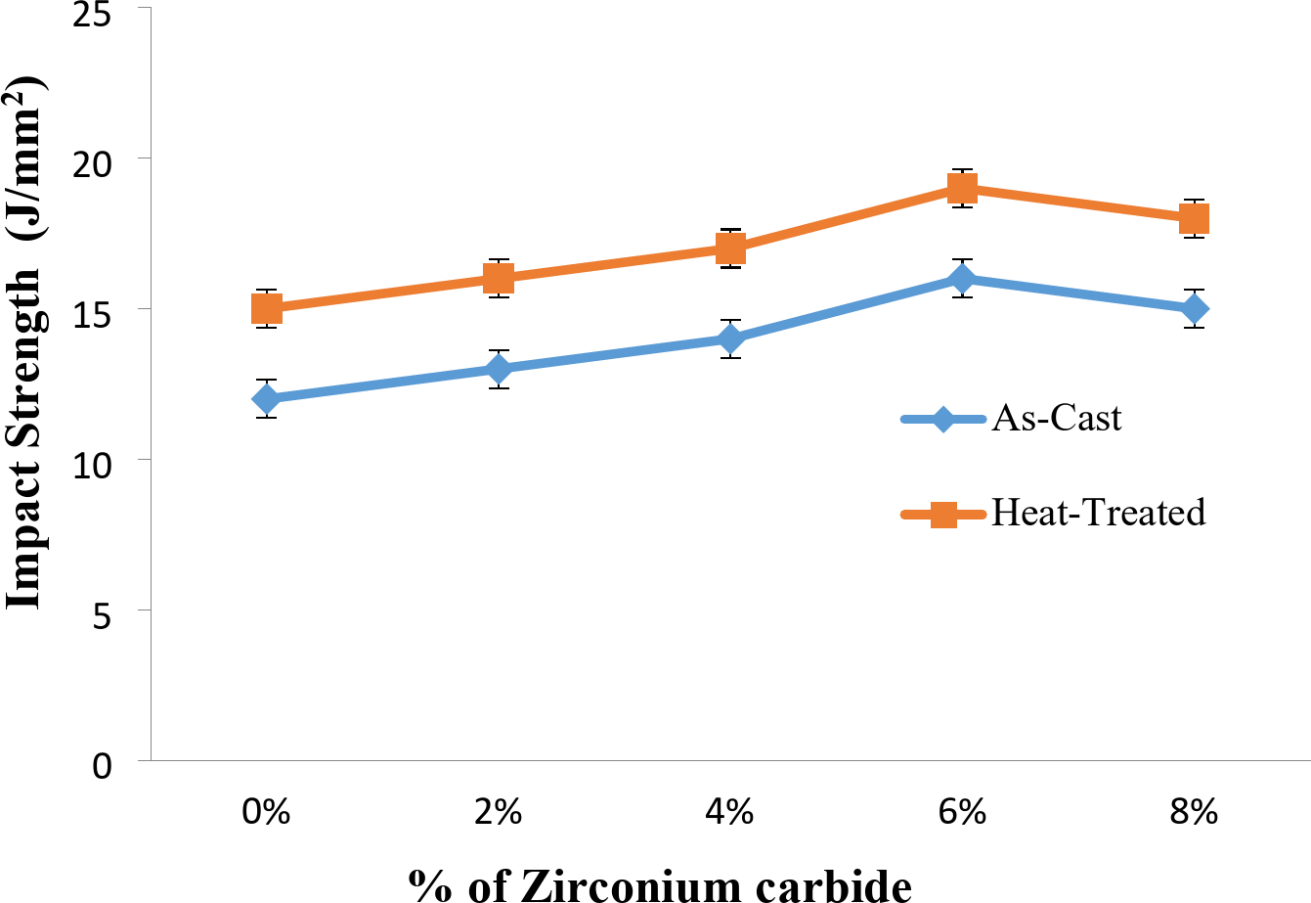
Impact strength vs. % of Zirconium Carbide of Al7475-ZrC Composites before and after heat treatment.

Figure 14 depicts the Impact strength vs. % of Zirconium Carbide of Al7475-ZrC Composites before and after the heat treatment. It is discovered that, the impact strength is more for heat treated Al7475-ZrC composite. Similar behavior has been observed by R. Vijayakumar^39^ et al. They have reported that, impact strength of heat-treated and water quenched Al6061-9%ZrO_2_ composite is optimum and is equal to 0.17 J/mm^2^.The heat-treated stir-cast Al7475-ZrC composites impact strength is higher than that of as-stir-cast composites^46^. Similar outcomes were attained by Raj Kumar et al.^15^. The heat-treated stir-cast Al7075 alloy-Nanographite-ZrO_2_ particles composites exhibited high impact strength in comparison to the Al7075-Nanographite-ZrO_2_ composites that have not been heat-treated. The impact strength values Al7075-Nanographite-ZrO_2_ composites were enhanced by 18% after heat treatment.

**Fig. 15:**
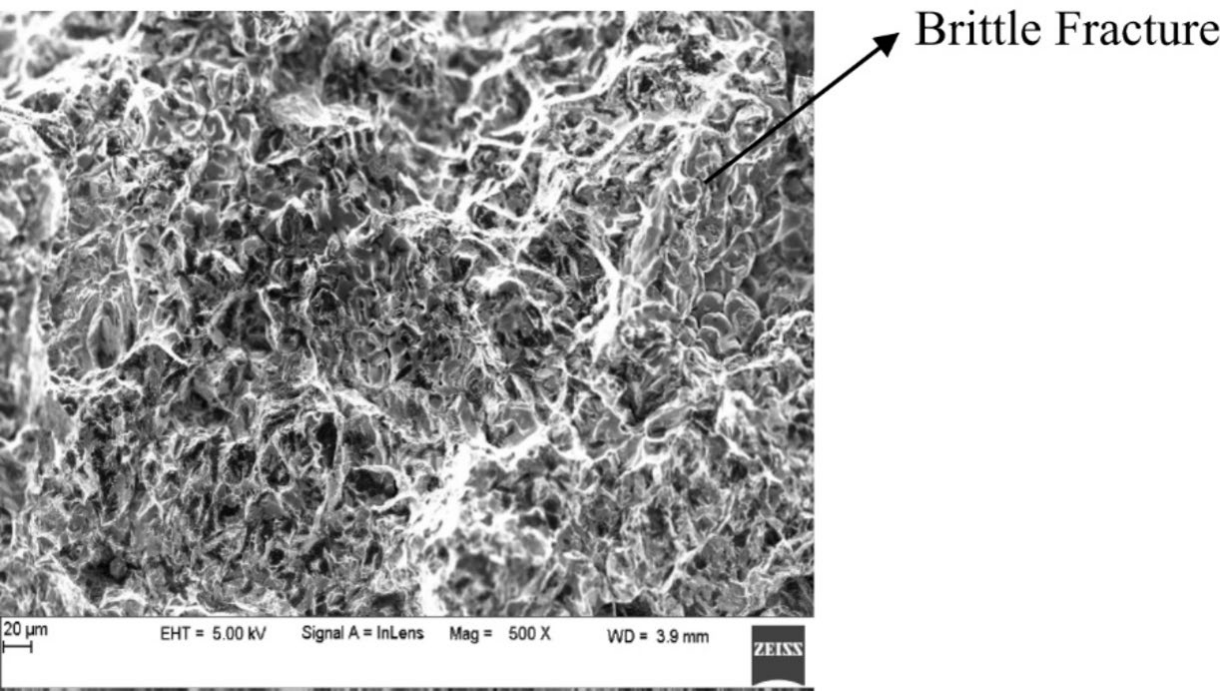
SEM fractograph of heat-treated stir-cast Al7475-6%ZrC composite.

The as-stir-cast Al7475-ZrC composite’s impact strength is 16 J/mm^2^, and that of heat-treated stir-cast Al7475-ZrC MMCs is 19 J/mm^2^. The Impact strength of heat-treated stir-cast composite is 16% more compared with as-stir-cast composite and 21% more compared with heat-treated stir-cast Al7475 alloy. The observed fracture due to impact is brittle in nature for both the heat-treated stir-cast as well as the as-stir-cast Al7475-ZrC composites. The SEM fractograph of heat-treated stir-cast Al7475-6%ZrC composite is shown in the Fig. 15.

## Conclusions

The results that can be inferred from the current examination on the effects of ZrC on the properties of as-stir-cast and heat-treated stir-cast Al7475-ZrC composites are as follows.

- The heat-treated stir-cast composite was characterized using scanning electron microscopy in which the micrograph indicates an even dispersion of ZrC particulates in the aluminum matrix.
- The mechanical properties of the T6 heat-treated stir-cast Al7475-ZrC MMCs are higher than the as-stir-cast composites. The mechanical characteristics are the highest for the heat-treated stir-cast Al7475-6% ZrC composite.
- The tensile strength and yield strength of heat-treated stir-cast Al7475-6%ZrC composite are 15% and 14% respectively higher than that of as-stir-cast Al7475-6%ZrC composite whereas the % elongation of heat-treated stir-cast Al7475-6%ZrC composite is 17% lower than the as-stir-cast Al7475-6%ZrC composite.
- The compression strength of heat-treated stir-cast Al7475-6%ZrC composite is 16% higher than that of as-stir-cast Al7475-6%ZrC composite.
- The hardness of heat-treated stir-cast Al7475-6%ZrC composite is 18% higher than that of as-stir -cast Al7475-6%ZrC composite.
- The impact strength of heat-treated stir-cast Al7475-6%ZrC composite is 16% more than the as-casted Al7475-6%ZrC composite and 21% more than the heat-treated stir-cast Al7475 alloy.
- The properties like UTS, compression strength, hardness and impact strength of Al7475-ZrC composite increases as the wt% of ZrC in the Al7475 matrix increases up to 6% of ZrC particles and further adding ZrC particles reduces the characteristics of the composites. Owing to the agglomeration of reinforcement particulates at 6% addition, clusters are formed in certain places.
- The developed Al7475-ZrC composites can further be investigated for wear behavior at different loads and speeds and also for optimization of wear process parameters by Taguchi technique. Al7475-ZrC composites can also be further investigated for corrosion and fatigue behavior.

## Data Availability

The datasets that were generated throughout the course of the current investigation can be obtained from the corresponding author, Subraya Krishna Bhat, upon making a fair request. All the data that was gathered for this study can be found within the article itself.
